# Mr. Stevenson on Cataract

**Published:** 1812-11

**Authors:** John Stevenson

**Affiliations:** Oculist and Aurist to her Royal Highness the Princess of Wales, and Member of the Royal College of Surgeons, &c. Great Russel-street, Bloomsbury-square


*For the Medical and Physical Journal.*

MR. STEVENSON *on* CATARACT.

(Continued from p. 265.)

ALLOW me next to offer a few practical remarks upon the Instruments I have found best adapted for operating upon the different kinds of Cataract. In the instance of Hannah Dove already detailed, and in several succeeding cases of firm lenticular, and dense and thickened membranous cataracts, I experienced considerable difficulty in my attempts with the needle of Mr. Saunders, freely to lacerate the tenacious capsules of the one, and to reduce to an adequate degree of comminution the hardened crystallines of the other species. These difficulties led me to believe, that the same form of instrument could not, with equal facility and success, be used for each description of disease; and that the needle itself, though much improved in its construction, did not appear to possess, even in its present shape, all the perfection of which it is susceptible. For, notwithstanding it penetrates the coats of the eye, and even a hard lens with ease, yet I found that under the latter circumstance it was apt, in consequence of being blunt above the angles, to become fixed and entangled in the cataract which, instead of yielding to, accompanied the several movements of the instrument, by adhering to its extremity. In consequence of that event, some danger was incurred of injuring the iris or ciliary processes, at the same time that little impression could be made upon the substance of the crystalline. It was probably in part owing to Mr. Saunders having met with similar impediments, that he subsequently preferred acting chiefly upon the *centre* of the *anterior lamella*

mella of the capsule, disregarding in a great measure, or only gently opening the texture of the lens in the first operation, until it had become softened by a partial solution in the aqueous humor. Although by that cautious method of procedure, there was undoubtedly less risk of inducing a hazardous inflammation; yet this advantage was obtained at the expense of a more tedious cure. Wishing to hasten the dissipation of the cataract by operating with greater freedom upon the crystalline and capsule at the same time, I gave directions to my instrument-maker (Mr. Ewing, Drury-lane,) upwards of eighteen months since, to construct one for the purpose of accomplishing my intentions with greater certainty and effect. As the needle alluded to differs in several respects from that of Mr. Saunders, I have taken the liberty of illustrating the particulars by the following correct description, and by the annexed faithful representation in a plate.

The blade of my needle is only eleven lines in length, (allowing twelve to the inch) one third of which from the shoulder downwards is round, and diminishes gradually from half to one third of a line in diameter. The remaining part of the instrument is gradually flattened to the extremity, which is very thin, flexible, and spear-pointed. Both the edges are rendered as sharp as possible to the extent of three lines above the angles, which are ground very obtuse, and where it is only one third of a line in breadth. From the point of the needle to its insertion into the handle it gradually increases in size, by which mechanism, not only is its introduction into the eye effected with less resistance, but likewise the escape of any portion of the aqueous or vitreous humor is at the same time altogether obviated. The handle is of the usual length, and octangular, and there are three dots on the side answering to the cutting edges as a guide to the operator, when the point of the instrument is concealed within the globe of the eye.

By shortening the needle in the way described, and which is still of a very sufficient length for all the purposes of the operation, more command is obtained over the motions of its point, for, the greater its length, the larger arc of a circle must it describe in the necessary movements within the organ of vision, and consequently the liability of wounding the delicate internal membranes of the eye must be proportionably increased. Besides which, the surgeon is not so capable, with a long needle, of accurately ascertaining how far it has penetrated the globe, or what degree of power he is exerting, before he has an opportunity of seeing it through the pupil, or when it happens to be suddenly obscured by the the effusion of the opake contents of a milky cataract. From experience too I learned, that the tendency to pain during the operation, and to inflammatory symptoms afterwards are counteracted, *cæteris paribus*, nearly in the ratio of the diminished size of the needle. Hence, mine is reduced to the greatest degree of tenuity of which it is susceptible, without hazard of breaking. On this account it requires, I must admit, a very steady hand, and a considerable share of boldness and decision, together with a practical acquaintance with the resistance that is usually opposed to the introduction of an instrument through the tunics of the eye, in order to prevent accidents.—It is, however, to the circumstance of its diminished size and peculiar form, that I am induced particularly to ascribe the very favorable result of my operations; as the unsuccessful issue of most of the cases operated upon formerly may, I apprehend, be in a great measure imputed to the ill-constructed and large size of the broad-spear-pointed couching needle employed by our ancestors (which, by not filling up the wound made by the spear part, facilitated the escape of the vitreous or aqueous humors, at the same time that, by the unnecessary breadth of its cutting edges, the iris was not unfrequently wounded) as to their unskilfulness in using it, and to their ignorance of the requisite degree of anatomical knowledge with respect to the structure of the eye, and especially with regard to the *posterior* chamber; about which the most dangerous, because the most vague and incorrect notions prevailed.

Against the slender form of the instrument here recommended, it has indeed been alleged, that it inflicts *not* an *incised* but a *punctured* wound; a species of injury unquestionably in some instances productive of alarming symptoms. This objection, however, by no means applies to the needle under consideration, which is so constructed as freely to *cut*, without in the slightest degree *contusing*, the parts through which it passes; and surely, where the great object is to secure re-union by the first intention, in order to excite as little irritation as possible, we cannot too studiously avoid unnecessarily enlarging the extent of the solution of continuity.—Finding likewise that the reduction of the lens and its capsule into small fragments, could with more certainty be effected by adopting Mr. Pott's method of "turning the instrument round and round between the finger and thumb within the body of the cataract, after its texture had been previously well loosened," in order more conveniently to adapt the needle to that purpose, it is *rounded*, instead of being flattened to some distance below the shoulder, and its angles also are rendered more obtuse.

The above-described needle, which I have now used during the space of a year and a half, with the most satisfactory result in lenticular cataracts, completely answers every purpose for which it was originally designed, as it not only readily penetrates, but also *cuts through* the diseased crystalline with great facility, so as to allow of its reduction into the smallest portions; by which its solution is proportionably accelerated.

As, however, the mode of using it differs considerably from that of the late Mr. Saunders, I think it right, in this place, to submit a concise description of the method to be pursued in performing the operation for *hard* lenticular cataract.

The patient having been properly seated, the pupil fully dilated by the *external* application of the belladonna, and the eye steadied by means of the fingers when practicable, or otherwise by a well-adapted speculum, the instrument is to be introduced in the usual manner through the sclerotica, at a distance not exceeding one line behind its junction with the cornea, with its flat side parallel to the plane of the iris. The needle is then to be carried to the front of the cataract, its point being projected across the anterior chamber to the nasal margin of the pupil. The cutting edge is next to be turned backward, when, by moving the needle so as to describe the segment of a circle, the capsule with the inclosed lens must be divided into nearly equal portions. Proceeding cautiously in a similar manner, by repeated transverse and perpendicular incisions, the whole crystalline and its enveloping membrane will be reduced into small flocculi. This object having been accomplished, the handle of the instrument is to be carefully rotated between the finger and thumb, by which manœuvre, the capsule is more effectually detached from its connections with the zona ciliaris at every point of its circumference, and the cohesion of the component parts of the lens more certainly destroyed. The whole, or as many of the fragments of the capsule and crystalline as the circumstances of the case will admit without danger of wounding the iris or ciliary processes, are then to be pushed forward into the anterior chamber, by means of the flat surface of the needle; after which it must be withdrawn in the same manner as it was passed into the globe of the eye.

By the above process, and by the complete central division of the cataract in the first instance, (a measure easily to be effected by the aid of my needle according to the foregoing directions,) the mischiefs arising from the unsupported lens revolving on the needle, and becoming partially protruded so as to press upon and overstretch the iris; or from its entire dislocation and subsequent escape through the pupil into the anterior chamber of the eye, are effectually obviated. The operation just detailed, is also with much greater justice and propriety entitled to the phrase "*radicitus tollit*" than either extraction or couching; since the possibility of the subsequent occurrence of secondary or membranous cataract is thereby prevented, as well as a radical cure eventually obtained, by the *entire removal of every part both of the capsule and lens.*—The dissipation of the disease in the above manner, requires a longer or shorter period to effect, according to existing circumstances; nor is it possible accurately to predict when that event will be fully accomplished, since various unforeseen causes may intervene to quicken or retard its completion. In a few instances I have known the cure obtained in the space of three or four weeks, whilst in other instances as many months have elapsed, and one or two repetitions of the process required, before the whole cataract has been absorbed.

I cannot however forbear observing in this place, that if Mr. Saunders was unnecessarily fearful "of pushing the fragments of the broken lens into the anterior chamber unless they tended that way," others seem to have carried the practice, without any modification or exception, to an opposite and far more dangerous extreme. That deservedly eminent surgeon Mr. Pott, though well aware that detached portions, and even the whole of the crystalline, when deposited in the anterior chamber (whether from their being there exposed to a larger quantity of its natural menstruum, or from the cataract being in that situation more completely separated from its connections, or from some other cause) disappeared with greater rapidity than when suffered to remain *in situ*, and accordingly practised what Scarpa so strongly inculcates at this day; yet still he thought the method wrong, not indeed from its inefficacy, but from an apprehension that it would be apt to occasion an irregularity of the pupil. Mr. Hey likewise, (Surgical Observations, p. 59 and 60,) who has seen, in several instances, the whole opake nucleus, and very often small opake portions, fall into the anterior chamber, makes the following excellent remark: "Indeed, if the cataract could in all cases be brought into the anterior chamber *without injury to the iris*, it would be the best method of performing the operation." From which passage it is evident that, in the opinion of this judicious practitioner, the above procedure with regard to the *displacement* of the ruptured lens (a term which, as Leveillé, the French translator of the Italian professor's valuable work on on the Eye, justly remarks, is very applicable to this mode of performing the operation), is *not in all* cases *safely practicable*. Candour however demands the concession, that the danger suggested by the distinguished writers just quoted, is, in a great measure, avoided in favorable cases, by availing ourselves of the peculiar influence of the belladonna. Still there are, according to my own observations, certain conditions of the organ of vision, which ought to restrain us from incautiously and rashly pushing even the broken lens with too much freedom into the anterior chamber: namely, 1st, when the pupil, from preceding inflammation of the iris, is more than usually contracted, and rendered incapable of being artificially dilated by the agency of the above-named narcotic; and 2dly, when the anterior chamber, on account of the flattened form of the cornea on the one side, or the protuberant state of the iris on the other, (from whatever causes arising,) happens to be uncommonly small in its dimensions. In the former example, there will be great risk, from an over officiousness to expedite the cure, by projecting the broken fragments of the hard lens through the diminished area of the pupil, of cutting or forcibly dilating the iris; either of which accidents may give birth to the most serious inconveniences. In the latter instance, the small quantity of the aqueous humor contained in the anterior chamber, could contribute in a very inconsiderable degree, to hasten the solution of the disorganized lens, beyond what it will spontaneously undergo, by being freely exposed to the action of that solvent in its natural situation. Whilst on the other hand the fragments, and still more the *nucleus* of a *hard* lens, (for so much of a *soft* one has sometimes escaped, or been pushed into the anterior chamber, as to fill this space even with the inferior edge of the pupil, giving the appearance of hypopium, with perfect impunity) confined between the iris and concave surface of the cornea, in highly susceptible states of the organ have, by their mechanical pressure or irritation, induced considerable pain, long continued inflammation, and eventually suppuration of the eye, and total loss of sight.

In the instances just stated, I greatly prefer Mr. Saunders's cautious method of suffering the ruptured lens to remain *in situ*, where I can assert, that I have never yet known a solitary example of its failing to become sooner or later dissolved, and so entirely absorbed, as not to leave the smallest vestige of a cataract behind. Nor is it thus circumstanced, (unless inadvertently made to press against the uvea, by which catastrophe a permanent dilatation, and possibly too an an irregularity of the pupil might ensue) disposed to excite any pain or inflammation. And surely it is better for the patient to be somewhat longer in obtaining a perfect restoration to sight, by our forbearing to do *too much*, than from an over anxiety on our part to accelerate the cure, to subject him to the dangers resulting from an injury of the iris. *Sat cito si benè sat.*—There is another consideration too that ought to reconcile the individual, laboring especially under the above mentioned complicated derangements, cheerfully to submit to this temporary retardation of his recovery; a suggestion with which Mr. Saunders perfectly coincided, viz. that his final restoration to sight will be thereby not only with greater certainty ensured, but likewise, by gradually regaining the use of his visual faculties in the way just stated supposing them to have been *long* lost, will probably be capable of enduring the full influence of light, and of freely exercising the organ nearly as soon as those persons who, after having been for a considerable period deprived of that blessing, have recovered it immediately upon the performance of an operation.

This opinion, however singular it may appear, I am induced to adopt from no inconsiderable experience and reflection; and the plausibility, if not the correctness of which, will receive some sanction from the following statements.

Mrs. C. (for many years an inmate in the Duke of Newcastle's family) upon whom I operated last year for reticulated capsular cataract, was enabled instantly after the needle was withdrawn to see very minute objects. The pain occasioned by the introduction of the instrument was very inconsiderable, and the slightest possible irritation supervened. The pupil, which before was so fixed and dilated that an eminent practitioner declined undertaking the operation, from an apprehension, I understood, that the above symptom was produced by, or complicated with, amaurosis, became at the same time in an highly active state. Although, since that period, no *visible* morbid affection has existed in any part of the organ, she has been capable only by slow degrees of bearing the stimulus of a strong light, or the exercise of reading or working.

Mr. Porter also, of Bethnal Green, was afflicted with a cataract in the left eye, of a description very similar to the preceding. I performed the operation in June last, with the effect of immediately restoring his lost sight; yet the same phenomena succeeded, and prevented him, till of late, from the comfortable use of the organ, notwithstanding the nicest examination could not detect the most trifling *apparent* defect, either in its external or internal organization; so far otherwise, otherwise, that I never recollect having seen an eye, after the most successful operation, which exhibited a more animated and healthful appearance.—The gradual manner therefore, in which patients recover by the diseased lens being put, by the method already detailed, into a condition fitted for spontaneous solution, so far from affording any valid objection against the operation suggested in these pages, (as the advocates for couching and extraction allege,) is, perhaps, a very strong recommendation in its favor. Do not reason indeed and experience concur in establishing the fact of the dangerous consequences which may and occasionally do actually arise, from the sudden and unguarded exposure of the retina to strong light, especially when it has acquired, in consequence of having been long subjected to a state of comparative darkness, (as is really the case, from the interception of the rays by the opake lens,) a high degree of accumulated sensorial power? And may not the intolerance of light, accompanied frequently with epiphora, and a contracted pupil subsequent to the operation of extraction, be in some instances fairly and reasonably imputed to this cause? The dangers alluded to may, it is true, be counteracted, by enjoining the patient to protect the organ for an adequate period of time, by means of an appropriate shade, so as *gradually* to accustom the eye to the full influence of light. But we cannot upon every occasion, expect to control his eager desire to enjoy the various delights and exquisite pleasures resulting from restored vision; the premature gratification of which I am persuaded, has not unfrequently laid the foundation of the formidable train of symptoms just enumerated.

But the above-described *straight* needle, although it proved admirably adapted for successfully operating upon simply *lenticular*, I found by no means equally well calculated for thickened and tenacious *capsular* cataracts; the usual elasticity and resistance of which, caused them to elude my efforts with that instrument, to tear or cut them into sufficiently small shreads for speedy solution in the aqueous humor. In cases of the latter description, I can succeed infinitely better by the aid of my *curved one-edge capsule*, or *iris knife*, which I directed to be made for the express purpose of operating upon Stephen Manbridge, a young man 17½ years of age, who was born with a congenital capsular cataract in each eye. This patient was brought to me last December, by Admiral Hamilton's son, from Boldre, New Forest, Hants. In this instance, such was the extreme tenacity and cohesion of the capsules, that I found myself incapable of effecting their necessary breach and free lace-

 ration, ration, by means of my straight needle. Thus foiled, I caused the instrument just mentioned to be constructed, of which the subjoined account affords a correct description, and the preceding plate an accurate delineation.

The blade of the knife is exactly one inch in length, half of which is straight, the remainder is slightly curved to the extremity. It cuts only on the concave edge from the point upwards to the extent of four lines, the opposite edge being blunt. From the point of the instrument to the middle of the blade, at which part it is only half a line in breadth, its thickness gradually increases; from whence, to the handle, it is conical, its diameter being progressively augmented from one-third to half a line. The handle is of the same length as that of the needle, and there are three dots corresponding with the cutting edge, and not any upon the reverse, a circumstance that should be constantly borne in mind by the operator, in order that he may be able to ascertain with certainty, the exact position of the instrument when its point is buried within the eye.

The pupil having been fully dilated by the belladonna, and all the previous steps of the operation having been duly arranged, I introduced the knife carefully through the coats of the eye at two lines behind the cornea, with its convexity forward. The cutting edge of the instrument being brought before the membranous cataract, its point was carried across the anterior chamber as far as the nasal edge of the pupil; when, by repeated cuts in different directions, I succeeded in reducing it to small filaments, and by cautious movements with the knife, was enabled to detach it completely from its connection with the ciliary zone except at one point, where a small triangular piece was left united by its basis to the hyaloid membrane, on the nasal side of each pupil. Part of the fragments fell into the anterior chambers, and in about ten weeks subsequently to the operation, the torn capsules became absorbed, leaving the whole ambit of the pupils in their natural state, beautifully transparent. When, however, the iris was unusually dilated, or when a lateral view was taken of the eyes, the adherent portions of the capsules became clearly visible. As generally happens under such circumstances, the fragments alluded to are probably by this time either wholly dissolved, or else so considerably contracted as in no degree to interfere with the functions of the organ. The iris in each eye was left in the most perfect and active condition, and vision rendered so complete, that he could discern any number of the smallest dots made with the point of a pen upon paper, and the quarters marked upon the dial of a watch with with the greatest ease and correctness, when aided by the assistance of proper convex glasses; the use of which were by no means required for any of the ordinary purposes of life. The extreme mildness of the process resorted to in the present instance, cannot be better illustrated than by stating, that he was regularly conducted on foot, immediately after each operation, from my house upwards of a mile, to his own lodgings; nor was he at any time confined to his room longer than two days, and not an hour to his bed.

Since the beginning of April, he has acted as servant to Mr. Hamilton, who with his accustomed humanity and benevolence, has had him taught the necessary duties of his situation; a species of knowledge he acquires, I understand, with considerable facility.—When he first obtained his sight, he judged very erroneously respecting distances, and the external physical properties of bodies, a defect in perception which he endeavored to correct by the powerfully auxiliary sense of touch. I do not recollect, however, that he described objects as appearing, to his apprehension, *inverted*. That irregular convulsive motion of the eyes, so peculiarly characteristic of *congenital* cataract accompanied with an opacity of the *whole capsules* of the lens, was improving when he left me; at which time he began to possess more command over the involuntary and unassociated actions of their muscles. Of his present state I cannot speak with precision, not having seen nor heard of him for several months.

In instances similar to the above where, from the operation having been so long postponed, the capsules have become unusually dense and tenacious, I have not the smallest hesitation in strongly recommending the employment of *my capsule knife*, as an instrument far preferable to any kind of *straight* needle hitherto invented. The instrument is likewise equally well adapted for the purpose of forming an *artificial pupil*, in cases requiring that operation; my method for accomplishing which, together with a description of an improved speculum, and further practical remarks on different points relative to the operation for cataract, must be reserved for a future occasion, having already exceeded the limits usually prescribed for medical communications.

I am, &c.

JOHN STEVENSON,

Oculist and Aurist to her Royal Highness the Princess of Wales, and Member of the Royal College of Surgeons, &c.

*Great Russel-street, Bloomsbury-square,*

